# Love chemistry

**DOI:** 10.1186/2041-2223-2-9

**Published:** 2011-04-04

**Authors:** Mark A Jobling

**Affiliations:** 1 Department of Genetics, University of Leicester, Leicester, LE1 7RH UK

## 

First impressions are supposed to count. The building in which I work is 1968 vintage and showing its age, but it was recently treated to a foyer makeover. These days, instead of the featureless entrance that we were accustomed to, you walk in through the sliding doors to be greeted by a floor-to-ceiling space-filling model of 60 base pairs of double-helical DNA, atmospherically lit from above by a hidden ring of blue lights. This is now a popular backdrop to photos of newly graduated students. The dominance of the iconic molecule of inheritance in a building occupied by health scientists and biologists, as well as geneticists, is partly explained by the plaque near the door, commemorating Alec Jeffreys' development of DNA fingerprinting, which took place here 26 years ago.

The plaque is actually a 'Chemical Landmark' [1] from the Royal Society of Chemistry. This, together with the tempting purple, red, white, black and blue plastic balls that represent the atoms of the double helix (nobody has stolen any of them yet, surprisingly), serves to remind us that beneath its glorious complexity, life is really chemistry.

Chemistry is chemistry, too, of course, and the department devoted to that particular subject has also had its foyer refreshed. Here there is a grand and stylish periodic table [2] on the wall, each element given its own perspex box, with most elements occupied by actual samples. Some scarce and highly radioactive elements are absent, and monochrome photographs of the great scientists after whom they are named peer out through the perspex as proxies. The hairy Dmitri Mendeleev himself, who is credited with the discovery of the periodic table, is among them, at element 101. If you stand back from the display, a few elements are particularly striking: the brilliant yellow powder of sulphur, the shining copper spheres, the vial of sticky brown bromine, the shimmering gold foil, the mercury, and the noble gases represented by discharge tubes formed into their chemical symbols, glowing eerily in sequence. But the general impression is of a lot of very similar looking, dull, silvery-grey metals with obscure names.

Similar they may look, but they all have different histories and characteristics. The story of how human curiosity and ingenuity has allowed us to understand the properties of the elements and exploit them in countless different applications is a rich and extraordinary one, and the best narrator I know is John Emsley, in his wonderful book *Nature's Building Blocks *[3]. The text is both encyclopaedic and engaging, listing the role of each element in biology and medicine, nutrition, war, the economy and the environment, as well as its chemical properties. Each also has an 'element of surprise' section: a quirky piece of information. My favourite is that for antimony: this has a powerful purgative effect, and Emsley tells us that in the past 'pills' of metallic antimony were used to relieve constipation and passed (quite literally) down from generation to generation for repeated use.

One element has emerged from obscurity recently in a most unwelcome way, making it onto prime-time news programmes. Emsley tells us that about 7,000 tonnes of zirconium are produced each year, and most of this is bought by the nuclear industry, since it is used to make the thousands of metres of casings for the uranium oxide fuel rods that power each nuclear reactor. It's ideal for this purpose, because it resists corrosion at high temperatures and does not absorb neutrons, and therefore fails to respond to the potent radioactivity of uranium by forming its own hazardous radioisotopes. Its close chemical cousin, hafnium, is found together with zirconium in nature. Hafnium absorbs neutrons very strongly, so the two must be separated with care before the zirconium tubes are made and deployed. When cooling failed at the Fukushima Daiichi nuclear plant in eastern Japan following the recent earthquake and tsunami, superheated steam reacted with hot zirconium to produce zirconium oxide and hydrogen, and when the hydrogen 'bubble' was vented it exploded, seriously damaging the reactor buildings. As a consequence of all this, more elements of the periodic table have emerged into the public arena. There is talk in the media of potassium iodide tablets being distributed and of possible threats from radioactive caesium and strontium.

The double helix in our foyer is no random 60 base pairs of sequence; it is, most appropriately, part of a minisatellite repeat array. It was 10 years after the Chernobyl accident of 1986 that these hypervariable markers were exploited by Yuri Dubrova and Alec Jeffreys [4] to demonstrate a significantly increased mutation rate in Belarusian children, arising from exposure of their parents to caesium-137. Currently there is optimism that Fukushima will not come to be remembered as a second Chernobyl. But the incident is a sharp reminder that our mastery of the periodic table's parade of elements, wonderful as it is, can have unpleasant side effects for the special bit of chemistry that is us.
